# Physiological Responses of *Serratia marcescens* to Magnetic Biochars and Coexisting Microplastics and the Relationships with Antibiotic Resistance Genes

**DOI:** 10.3390/toxics14010035

**Published:** 2025-12-28

**Authors:** Guixiang Zhang, Rui Ren, Xiaohui Zhang, Yuen Zhu, Yanxia Li, Long Ping

**Affiliations:** 1 School of Environment and Resources, Taiyuan University of Science and Technology, Taiyuan 030024, China; 2 Engineering Research Center of Coal-Based Ecological Carbon Sequestration Technology of the Ministry of Education, Key Laboratory of Graphene Forestry Application of National Forest and Grass Administration, Shanxi Datong University, Datong 037009, China; 3 Shanxi Laboratory for Yellow River, College of Environmental & Resource Sciences, Shanxi University, Taiyuan 030006, China; 4 State Key Laboratory of Water Environment Simulation, School of Environment, Beijing Normal University, Beijing 100875, China

**Keywords:** magnetic biochars, *Serratia marcescens*, microplastics, physiological characteristics, antibiotic resistance genes

## Abstract

Magnetic biochars (MBCs) have been shown to inhibit the horizontal transfer of antibiotic resistance genes (ARGs) in soils, both with and without microplastics (MPs); however, the underlying molecular biological mechanisms remain unclear. This study examined the effects of MBCs and coexisting polybutylene adipate terephthalate microplastics (PBAT MPs) on the physiological characteristics of *Serratia marcescens* ZY01 (a host strain carrying the *tet* gene) and further investigated their relationships with the absolute abundance of the *tet* gene in soil. The results demonstrated that MBCs promoted prodigiosin synthesis in *Serratia marcescens* ZY01 by mediating the electron transfer process, the effect of which was further enhanced in the presence of PBAT MPs. In treatments without PBAT MPs, MBCs generally suppressed the production of both proteins and polysaccharides in the extracellular polymeric substances. In contrast, in treatments containing PBAT MPs, the protein content gradually decreased with decreasing iron-to-biochar ratios, while the polysaccharide content remained largely unchanged. MBCs also elevated intracellular ROS levels due to the increased oxidative stress, particularly in treatments with PBAT MPs. A positive correlation between intracellular ROS levels and cell membrane permeability indicates that intracellular ROS was the primary driver of the increased cell membrane permeability. The presence of MBCs and PBAT MPs generally provided favorable habitats for *Serratia marcescens* ZY01, thereby enhancing its cell viability. Mantel test analysis indicated that MBCs influenced *Serratia* growth in soil by modulating its cell viability. Furthermore, the increased intracellular ROS level was significantly positively correlated with the absolute abundance of the *tet* gene in soil, implying the horizontal transfer of the *tet* gene at the intra-genus level. These findings offer helpful insights for developing environmental remediation strategies based on biochar–iron composites.

## 1. Introduction

Antimicrobial resistance genes (ARGs) are posing a major global health threat, already causing approximately 5 million deaths in 2019 and 8 million deaths annually by 2050 [1]. As a pathogenic member of *Enterobacterales*, *Serratia marcescens* not only causes urinary tract, respiratory tract infections and bacteremia, but also exhibits an exceptional proficiency in acquiring, transferring, and modifying the expression of ARGs [1,2]. Of particular concern, *Serratia marcescens* has been detected in various environmental matrices worldwide, with a notable proportion isolated from soil [2]. The challenging issue of soil contamination by antibiotic resistance microorganisms like *Serratia marcescens* and ARGs is increasing worldwide.

Various factors can influence the dissemination of ARGs in soil, such as coexisting contaminants and microbial community succession [3,4]. For example, microplastics (MPs) as emerging contaminants in soil can drive the enrichment of ARGs in soil not only by promoting horizontal gene transfer via enhancing ARG and mobile genetic element (MGE) co-occurrence, but also by enriching ARG hosts such as Proteobacteria [5]. The coexistence of *Serratia marcescens* with MPs has been shown to enhance the horizontal transfer of the *tet* gene in soil, a process driven dominantly by the enrichment of specific Actinobacteriota genera [6]. Besides the enrichment of ARG host bacteria, the dissemination of ARGs is also closely linked to the physiology of bacterial cells, such as the fluctuations in reactive oxygen species (ROS), the alterations in cell membrane permeability, the secretion of extracellular polymeric substances (EPSs), and the shifts in energy metabolism [7,8]. For example, MPs and their released exogenous additives synergistically enhanced the conjugative transfer of ARGs by increasing ROS levels and cell membrane permeability [9,10]. The outer membrane proteins also play an important role in the dissemination of ARGs by enabling recognition and interaction between their bacterial hosts [3]. It has been demonstrated that the presence of MPs can facilitate the gene expression levels of outer membrane proteins and accelerate the formation of conjugative pairing systems, thereby enhancing the conjugative transfer of ARGs among microorganisms [11]. Therefore, effective control strategies are urgently needed to prevent the dissemination of ARGs in soil environments, particularly in soil coexisting with MPs.

Biochar, a carbon-rich material derived from the pyrolysis of biomass in oxygen-limited environments, is highly valued for its diverse agricultural benefits, such as improving soil fertility, boosting crop yield, enhancing nutrition use efficiency, and aiding in the management of plant disease and contamination stress [12]. Although biochar treatment was shown to reduce 12.1% of ARGs host bacteria in soil [13], some other studies reported that biochars can increase the relative abundance of ARGs due to the variations in biochar type and the presence of coexisting contaminants [14,15]. To address this limitation of original biochar on reducing ARGs in soil, modified biochars have been explored to inhibit the dissemination of ARGs. Among them, the application of biochar–iron composites has been considered as a promising strategy that is synergistic, efficient, economical, and eco-friendly [16]. For example, nano zero-valent iron (nZVI)-modified biochar reduced the relative abundance of ARGs through regulating extracellular and intracellular ROS levels [17]. nZVI also inhibited the horizontal transfer of ARGs by reducing EPS content [18]. However, nZVI exhibited strong toxicity to soil organisms when it coexisted with organic contaminants [19]. Compared with nZVI-modified biochar, magnetic biochar is more environmentally friendly due to its reliance on non-radical pathways for environmental remediation [20]. Furthermore, it has been demonstrated that magnetic biochars effectively reduce over 60% of tetracycline resistance gene (*tet*) in soil without biodegradable polybutylene adipate terephthalate (PBAT) MPs and also effectively suppress the dissemination of *tet* in soil even with coexisting MPs [21]. A recent study also found that MgFe_2_O_4_-loaded biochar could inactivate antibiotic resistance microorganisms and degrade ARGs by damaging cell membranes, proteins, and lipids via increasing intracellular ROS levels [22]. However, it remains unclear how magnetic biochars inhibit the dissemination of ARGs in soil by regulating physiological characteristics of its host bacterial cell, particularly in the condition of coexisting MPs.

In this study, we isolated and identified a bacterial strain (*Serratia marcescens* ZY01 carrying the *tet* gene) to investigate the effects of magnetic biochars on its physiology and their relationships with the dissemination of the *tet* gene in soil with and without PBAT MPs. The variation of key physiological parameters of *Serratia marcescens* after the addition of magnetic biochars, including prodigiosin synthesis, protein and polysaccharide contents in EPS, intracellular ROS level, cell membrane permeability, and cell viability, was assessed in the systems with and without PBAT MPs. Furthermore, we discussed the role of magnetic biochars in regulating the physiology of *Serratia marcescens* and the consequent dissemination of the *tet* gene in soil with and without PBAT MPs. The results of this study provide valuable insights into the application of magnetic biochars to remediate soil contaminated with antibiotic resistance genes (ARGs), particularly in soil coexisting MPs.

## 2. Materials and Methods

### 2.1. Chemicals and Materials

The solution of 3-(4,5-Dimethyl-2-thiazol)-2,5-diphenyltetrazolium bromide (MTT) was purchased from Beijing Solarbio Science & Technology Co., Ltd. (Beijing, China). The lactate dehydrogenase (LDH) detection kit was obtained from the Nanjing Jiancheng Institute of Bioengineering (Nanjing, China). Other biochemical reagents, including reactive oxygen species (ROS) kit and 2′,7′-dichlorofluorescein diacetate (DCFH-DA), were supplied by Shanghai Beyotime Biotechnology Co., Ltd. (Shanghai, China). All other chemicals, such as K_2_HPO_4_, KH_2_PO_4_, NaCl, concentrated H_2_SO_4_, phenol, and glucose, were of reagent grade and used as received. The phosphate-buffered saline (PBS) solution (1L, pH 7.2) was prepared with ultrapure water containing NaCl (8 g), K_2_HPO_4_ (1.15 g), KH_2_PO_4_ (0.2 g), followed by sterilization at 121 °C under high pressure for 15 min.

The PBAT MPs were purchased from Shandong Yusuo Chemical Technology Co., Ltd. (Linyi, China) and sieved to a uniform size of <154 μm (100-mesh). A bacterial strain carrying the *tet* gene was isolated from sewage sludge using LB medium containing oxytetracycline (100 µg/mL). This strain was identified as *Serratia marcescens* and designated as *Serratia marcescens* ZY01. The surface soil (0–20 cm) was collected from a site near the Dongshan Campus of Shanxi University. The soil was classified as loamy sand (89.59% sand, 8.85% silt, and 1.56% clay). It had a pH of 7.98 (soil/water = 1:2.5, *w*/*v*) and an organic matter content of 4.38 g/kg.

### 2.2. Preparation and Characterization of Magnetic Biochars

The methods for preparing and characterizing the magnetic biochars (MBCs) have been described in our previous study [21]. Briefly, MBCs were synthesized from corncob biochar (BC), FeCl_3_·6H_2_O, and FeCl_2_·6H_2_O via a wet co-precipitation method. The resulting MBCs were labeled as MBC31, MB21, MBC11, MBC12, MBC13, MBC15, and MBC17 based on the iron-to-biochar mass ratios of 3:1, 2:1, 1:1, 1:3, 1:5, and 1:7, respectively. These ratios were selected based on their different electron transfer capacities, which were a key factor influencing the physiology of *Serratia marcescens* ZY01. The obtained MBCs were characterized for their surface morphology, specific surface area, surface elemental composition, iron valence state distribution, and electrochemical impedance spectroscopy.

### 2.3. Experiment on Effects of MBCs and PBAT MPs on Physiology of Serratia marcescens ZY01

#### 2.3.1. Culture Treatments of *Serratia marcescens* ZY01

After activation of *Serratia marcescens* ZY01 in the sterile LB medium (30 °C, 150 rpm), 10% (*v*/*v*) of inoculum was transferred to the fresh LB medium and incubated under the same conditions for 12–18 h. The cells were then harvested by centrifugation, washed, and resuspended in the sterile PBS to an OD_600_ of 1.0 for subsequent experiments. All MPs and MBCs were sterilized by UV irradiation for 3 h before use. Experimental treatments were set up in two groups: (1) The treatments without PBAT MPs, where BC/MBCs (1 wt%) were separately mixed with the bacterial suspension. The treatment labels matched those of the added materials. (2) The treatments with PBAT MPs, where BC/MBCs (1 wt%) and PBAT MPs (1 wt%) were co-introduced into the bacterial suspension. These treatments were labeled as BCP, MBC31P, MBC21P, MBC11P, MBC12P, MBC13P, MBC15P, and MBC17P. All mixtures were agitated vigorously and then incubated on a shaker (30 °C, 150 r/min) for 72 h.

#### 2.3.2. Determination of Physiological Indicators of *Serratia marcescens* ZY01

After incubation, the above mixture of bacterial suspensions and materials was analyzed for the following physiological indicators of *Serratia marcescens* ZY01, including prodigiosin, protein and polysaccharide contents in EPS, intracellular ROS level, cell membrane permeability, and cell viability. The specific methods were conducted as follows.

Prodigiosin was extracted by adding 0.5 mL of the above mixture to 4.5 mL of methyl alcohol (pH = 3.0), keeping for 30 min. After centrifugation, the supernatant was collected and the prodigiosin content was measured at 534 nm using a microplate reader. The relative yield level of prodigiosin was expressed as the ratio of the absorbance intensity of prodigiosin from the treatments with materials to that from the control group (*Serratia marcescens* ZY01 alone).

The protein content in EPS: The mixture was centrifuged, and the pellet was resuspended to its original volume with 0.9% of NaCl. After shaking (80 r/min, 1 min) and centrifugation (8500 r/min, 15 min, 4 °C), the supernatant was filtered (0.45 µm) to obtain the loosely bonded EPS (LB-EPS). The resuspended LB-EPS in 0.9% of NaCl was heated at 60 °C for 30 min, centrifuged (9000 r/min, 20 min, 4 °C), and filtered (0.45 µm) to obtain the tightly bonded EPS (TB-EPS). Coomassie Blue Solution (0.5 mL, 100 mg of Coomassie Blue in water with 50 mL of 95% ethanol and 100 mL of 85% phosphoric acid) was added into the TB-EPS solution (1 g/L, 0.1 mL), keeping for 2 min. The protein content was measured at 595 nm using a fully automatic microplate reader.

The polysaccharide content in EPS: The EPS solution (1 g/L, 0.1 mL) was diluted to 1.0 mL with ultrapure water, mixed with 1 mL of 5% phenol solution, and then 5 mL of concentrated sulfuric acid was added. After incubation for 5 min, the sample was heated at 96 °C for 20 min in the dark. After cooling rapidly to room temperature, the absorbance was measured at 490 nm/485 nm. The standard curve was prepared using glucose after extraction and determination as the above method.

The intracellular ROS level: The mixture was filtered (10 μm of pore size) to obtain a bacterial suspension, which was centrifuged (10,000 r/min, 3 min) and washed three times using 0.9% of NaCl. The intracellular ROS levels were determined using 2′,7′-dichlorofluorescein diacetate (DCFH-DA) according to the kit instructions. The obtained results were expressed as the fluorescence intensity ratio relative to the control (*Serratia marcescens* ZY01 alone).

The cell membrane permeability: The lactate dehydrogenase (LDH) activity in the bacterial suspension was measured at 450 nm following the kit protocol. Cell membrane permeability was expressed as the ratio of LDH activity in the treatments with materials to that in the control (*Serratia marcescens* ZY01 alone).

The cell viability: The mixture (OD_600_ = 1.0, 1 mL) was mixed with 0.2 mL of MTT solution. After incubation at 37 °C for 60 min and centrifugation (12,000 r/min, 10 min), the formazan precipitate was dissolved in the dimethyl sulfoxide. The absorbance was measured at 510 nm and 690 nm. The cell viability was calculated as OD_510_ minus OD_690_ to correct for material turbidity.

### 2.4. Soil Incubation and Analysis

The soil incubation was conducted as previously described [21], with the detailed analytical methods for the *tet* gene and *Serratia* abundance therein. Briefly, 10 mL of the resuscitated *Serratia marcescens* ZY01 (OD_600_ = 1) was inoculated into the soil (990 g) containing 1 mg/kg of oxytetracycline. After aging in darkness for one week, PBAT MPs (1%, *w*/*w*) were added to the designed treatments. Then, BC/MBCs (1%, *w*/*w*) were separately incorporated into soils with and without PBAT MPs. All soil mixtures were homogenized and incubated in the dark at 20 °C for 30 days, maintaining 70% water-holding capacity. Upon completion, the fresh soil samples were destructively collected for further analysis. The absolute abundance of the *tet* gene was detected and quantified by quantitative polymerase chain reaction (qPCR, ABI7500, Thermo Fisher Scientific Co., Ltd., New York, NY, USA). The relative abundance of *Serratia* in soil was determined using high-throughput sequencing.

### 2.5. Data Analysis

A one-way analysis of variance (ANOVA) was employed to analyze the statistically significant differences between different treatments using SPSS 27 software, followed by Tukey’s post hoc test (with significance levels at *p* < 0.05, *p* < 0.01, and *p* < 0.001). Data visualization, including the creation of bar charts, scatter plots, and regression fits, was performed using Origin 2024. Additionally, a Mantel test was conducted and its results visualized with the Chiplot tool (https://www.chiplot.online).

## 3. Results

### 3.1. Correlations Between Serratia and tet Gene in Soil

**Figure 1:**
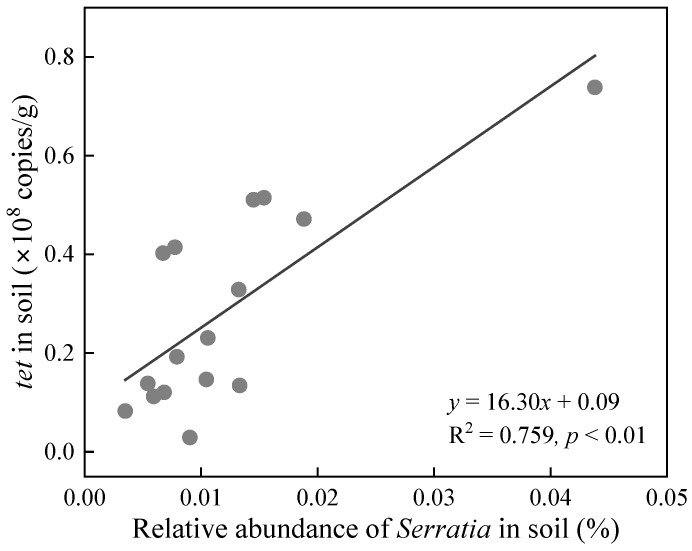
Correlation between relative abundance of *Serratia* and absolute abundance of *tet* in soil.

As reported in our previous study [21], BC/MBCs influenced the relative abundance of *Serratia* (Figure S1) and the absolute abundance of the *tet* gene (Figure S2) in soil with and without PBAT MPs. Briefly, BC, MBCs, and PBAT MPs each significantly reduced the relative abundance of *Serratia* in soil. Compared with the soil with PBAT MPs, BCP and MBCP treatments generally did not significantly change the relative abundance of *Serratia* in soil, with the exception of the MBC15P treatment. Although MBCs effectively suppressed the dissemination of the *tet* gene in soils without PBAT MPs (efficiency > 60%), their impact was minimal in soil with PBAT MPs (except for the MBC15 treatment). As shown in Figure 1, there was a significantly positive correlation between the relative abundance of *Serratia* and the absolute abundance of the *tet* gene in soils, indicating the key role of *Serratia* in the enrichment of the *tet* gene in the studied soil. Tetracycline resistance (*tet*) genes are commonly detected in the bacterial genus belonging to *Enterobacterales* [23], such as *Serratia marcescens* ZY01 in this study.

### 3.2. Effects of BC/MBCs and PBAT MPs on Physiological Characteristics of Serratia marcescens ZY01

**Figure 2:**
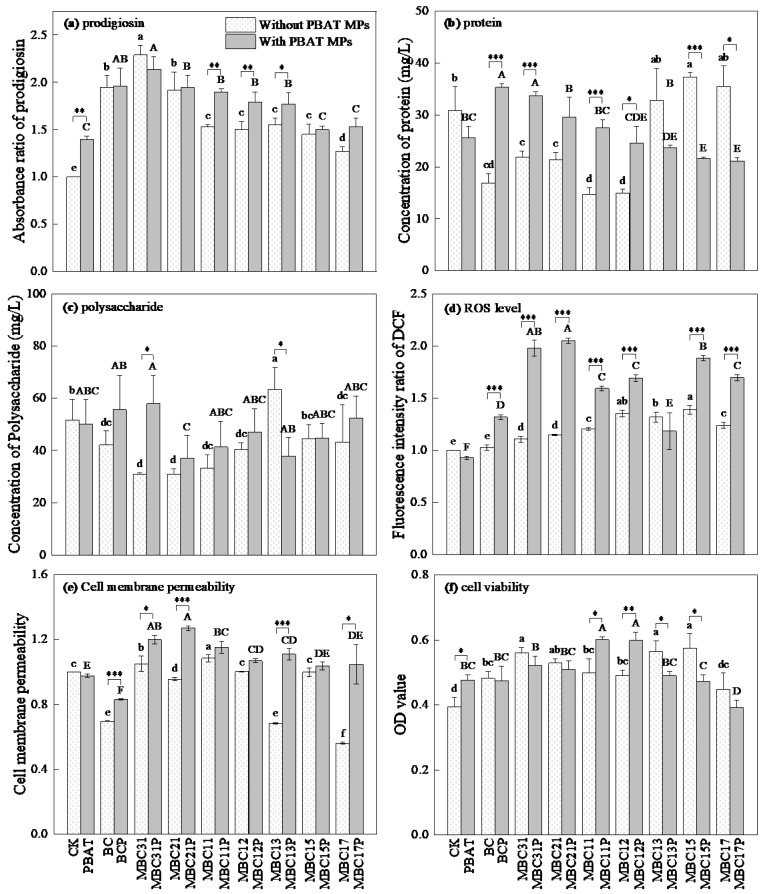
Effects of BC/MBCs and PBAT MPs on physiology characteristics of *Serratia marcescens* ZY01 (The different lowercase letters mean significant differences between different treatments without PBAT MPs, *p* < 0.05. The different uppercase letters mean significant differences between different treatments with PBAT MPs, *p* < 0.05. The asterisks “*, **, and ***” represent the significant difference at *p* < 0.05, *p* < 0.01, and *p* < 0.001, respectively).

As illustrated in Figure 2a, all biochar-based materials significantly increased prodigiosin production in the PBAP MP-free treatments (*p* < 0.05), with efficacy varying by the iron-to-biochar ratio. Compared with the control treatment (CK), the presence of PBAT MPs significantly promoted prodigiosin synthesis by *Serratia marcescens* ZY01 (*p* < 0.05). Furthermore, in the treatments with PBAT MPs, the addition of BC and most MBCs (excluding the MBC15P and MBC17P treatments) led to a further significant increase in prodigiosin synthesis (*p* < 0.05). Importantly, when the iron-to-biochar ratios of MBCs ranged from 1:1 to 1:3, the prodigiosin yields were significantly higher in the treatments with PBAT MPs than those in the treatments without PBAT MPs (*p* < 0.05 or *p* < 0.01).

The protein content in the extracellular polymeric substance (EPS) was significantly altered by the addition of BC/MBCs into the treatments with and without PBAT MPs (Figure 2b). In the treatments without PBAT MPs, BC and MBCs with higher iron-to-biochar ratios (3:1–1:2) significantly decreased the protein content in EPS (*p* < 0.05), whereas MBC15 significantly increased it (*p* < 0.05). The PBAT MPs alone had no significant effect on the protein content in EPS (*p* > 0.05). In the treatments with PBAT MPs, both BCP and MBC31P treatments increased the protein content in EPS compared with the PBAT MP-only treatment (*p* < 0.05), while MBC13P, MBC15P, and MBC17P significantly decreased it (*p* < 0.05). Furthermore, the protein contents in the BCP, MBC13P, MBC11P, and MBC12P treatments were significantly higher than in their corresponding treatments without PBAT MPs (*p* < 0.001 or *p* < 0.05). Conversely, MBC15P and MBC17P treatments showed significantly lower protein contents in EPS compared with the MBC15 and MBC17 treatments (*p* < 0.001 and *p* < 0.05, respectively).

Regarding the polysaccharide content in EPS (Figure 2c), BC and most MBCs (except for MBC13 and MBC15) significantly decreased its content (*p* < 0.05). In contrast, MBC13 significantly increased the polysaccharide content in EPS (*p* < 0.05), while MBC15 showed no significant effect on it (*p* > 0.05). Similar to the trend observed for the protein content in EPS, the treatment with PBAT MPs alone did not significantly influence the polysaccharide content in EPS (*p* > 0.05). However, the polysaccharide content in the MBC31P treatment was significantly higher than that in the MBC31 treatment (*p* < 0.05), while the opposite was true for the MBC13P treatment compared with the MBC13 treatment (*p* < 0.05).

The intracellular ROS levels in *Serratia marcescens* ZY01 are shown in Figure 2d. In the treatments without PBAT MPs, all MBCs significantly elevated ROS levels compared with the CK treatment (*p* < 0.05), with the highest level observed in the MBC15 treatment. The treatment with PBAT MPs alone did not significantly alter the intracellular ROS levels. However, all MBCP treatments significantly increased the intracellular ROS levels compared with the treatment with PBAT MPs alone (*p* < 0.05), with the MBC31P and MBC21P treatments showing the most pronounced effects. Moreover, the intracellular ROS levels in all PBAT MP-containing treatments were generally higher than those in their corresponding PBAT MP-free treatments, except for MBC13 and MBC13P.

The effects of BC/MBCs on the cell membrane permeability of *Serratia marcescens* ZY01 were highly dependent on the iron-to-biochar ratios (Figure 2e). In treatments without PBAT MPs, BC, MBC21, MBC13, and MBC17 significantly decreased the cell membrane permeability of *Serratia marcescens* ZY01 (*p* < 0.05), whereas MBC31 and MBC11 significantly increased it (*p* < 0.05). The MBC12 and MBC15 treatments had no significant effect on its cell membrane permeability (*p* > 0.05). The treatment with PBAT MPs alone also caused no significant change in it (*p* > 0.05). In the treatments with PBAT MPs, BCP treatment significantly decreased the cell membrane permeability of *Serratia marcescens* ZY01 (*p* < 0.05), while most MBCP treatments (excluding MBC15P and MBC17P treatments) significantly increased it (*p* < 0.05). Furthermore, the cell membrane permeability of *Serratia marcescens* ZY01 in BCP, MBC31P, MBC21P, MBC13P, and MBC17P treatments was significantly higher than that in their corresponding PBAT MP-free counterparts (*p* < 0.001, *p* < 0.01, or *p* < 0.05).

As presented in Figure 2f, BC and most MBC treatments (except for the MBC17 treatment) significantly increased the cell viability of *Serratia marcescens* ZY01 compared with the CK treatment (*p* < 0.05), with the highest level observed in the MBC31, MBC13, and MBC15 treatments. The treatment with PBAT MPs alone also significantly enhanced the cell viability of *Serratia marcescens* ZY01 compared with the CK treatment (*p* < 0.05). In the treatments containing PBAT MPs, the MBC11P and MBC12P treatments significantly increased the cell viability of *Serratia marcescens* ZY01 compared with the PBAT MPs alone treatment (*p* < 0.05), whereas the MBC17P treatment significantly decreased it (*p* < 0.05); other MBCP treatments showed no significant effect on it (*p* > 0.05). Direct comparisons between the treatments with and without PBAT MPs revealed that the cell viability of *Serratia marcescens* ZY01 was significantly higher in the MBC11P and MBC12P treatments than in the MBC11 and MBC12 treatments (*p* < 0.05 or *p* < 0.01). In contrast, the cell viability of *Serratia marcescens* ZY01 in the MBC13P and MBC15P treatments was significantly lower than in the MBC13 and MBC15 treatments (*p* < 0.05).

### 3.3. Effects of the Changes in Physiological Characteristics of Serratia marcescens ZY01 on Serratia and tet Gene Abundances in Soil

**Figure 3:**
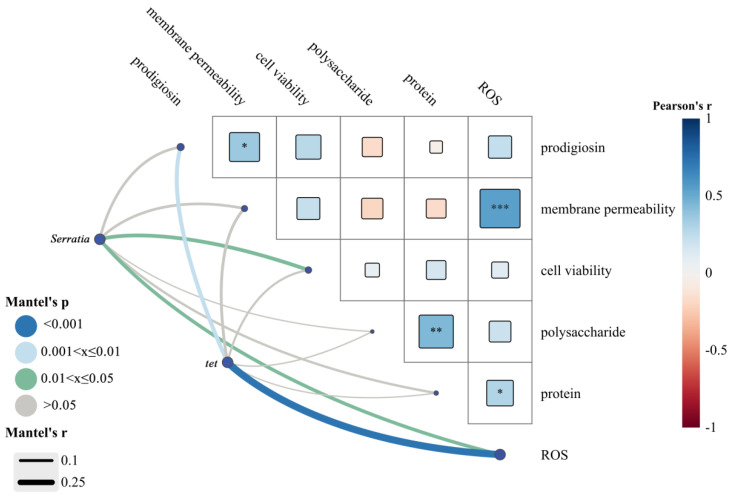
Mantel test analysis between the relative abundance of *Serratia* in soil, the absolute abundance of the *tet* gene in soil, and the physiological characteristics of *Serratia marcescens* ZY01 (The asterisks “*, **, and ***” represent the significant difference with *p* < 0.05, *p* < 0.01, and *p* < 0.001, respectively).

A Mantel test analysis was performed to evaluate the correlations between the relative abundance of *Serratia* in soil, the absolute abundance of the *tet* gene in soil, and the physiological characteristics of *Serratia marcescens* ZY01 (Figure 3). The relative abundance of *Serratia* in soil showed significantly positive correlations with the cell viability of *Serratia marcescens* ZY01 and the intracellular ROS levels (*p* < 0.05). Similarly, the absolute abundance of the *tet* gene in soil was positively correlated with the prodigiosin synthesis (*p* < 0.01) and intracellular ROS levels (*p* < 0.001). Among the physiological parameters, the prodigiosin synthesis was significantly positively correlated with the cell membrane permeability of *Serratia marcescens* ZY01 (*p* < 0.05). The intracellular ROS levels exhibited significantly positive correlations with the cell membrane permeability of *Serratia marcescens* ZY01 (*p* < 0.001) and the protein content in EPS (*p* < 0.05). Additionally, a significantly positive correlation was observed between the protein and polysaccharide contents in EPS (*p* < 0.05).

## 4. Discussion

Our previous work demonstrated that, in soils with and without PBAT MPs, MBCs effectively inhibited the horizontal transfer of the *tet* gene between different bacterial genera at the inter-genus level in soil. This inhibition was linked to a reduction in the relative abundance of degradation/utilization/assimilation metabolic pathways in the soil bacterial community [21]. To further elucidate the underlying microbiological mechanisms at the physiological level, it is essential to examine the physiological changes of *Serratia marcescens* ZY01 after exposure to BC/MBCs and the coexistence of PBAT MPs.

The prodigiosin, a secondary metabolite produced by *Serratia marcescens*, is known to form strong iron chelates [24,25]. Thus, the addition of MBCs likely stimulated the production of prodigiosin in *Serratia marcescens* ZY01 because of the increase in iron contents in the systems. Furthermore, the prodigiosin itself is an electrochemically active substance [26]. Therefore, BC/MBCs as an electron mediator to regulate electron transfer process [25,27] explained their stimulatory effect on the prodigiosin synthesis in *Serratia marcescens* ZY01. A recent study also demonstrated that MBCs enriched electroactive bacteria and boosted electron transfer [28]. As shown in Figure 2a, the extent of stimulation depended on the iron-to-biochar ratios of MBCs, possibly because iron could play a role in preserving the redox potential of prodigiosin [29]. Although PBAT MPs also stimulated the prodigiosin synthesis, the underlying mechanisms might be different from the BC/MBCs treatments. The prodigiosin synthesis is believed to occur on the cell membrane, because *PigC* encoding, a key enzyme involved in the final step of its synthesis, is located on the cell membrane [30]. MPs have been reported to cause cell membrane damage, including lower density, fluidity changes, and membrane thickening [31], which may explain their trigger effect on prodigiosin synthesis. When BC/MBCs were added to the treatments containing PBAT MPs, the prodigiosin synthesis further increased compared with treatment with PBAT MPs alone, indicating a critical role of electron mediation in regulating prodigiosin synthesis. Notably, MBCs with iron-to-biochar ratios of 1:1–1:3 showed a significant greater enhancement of prodigiosin synthesis when the treatments coexisted with PBAT MPs. This finding may be attributable to the interactions between MBCs and PBAT MPs. MBCs with iron-to-biochar ratios of 1:1–1:3 exhibited stronger electron transfer capacity than other MBCs, which could more effectively enhance the degradation of organic compounds [21]. Consequently, these MBCs may more strongly alter the surface functional groups on PBAT MPs via enhancing their degradation, which further strengthens the electron transport chain and boosts energy supply in the treatments, ultimately leading to the enhanced prodigiosin synthesis.

The production of both protein and polysaccharide in EPS by *Serratia marcescens* ZY01 was generally inhibited by BC and most MBCs (excluding those with lower iron-to-biochar ratios). This aligns with reports that the conductive materials can alter the EPS composition [32]. For example, biochar and granular-activated carbon decreased EPS content, while nano zero-valent iron (nZVI) and magnetite increased it. Another study reported that soil microbe cultivation with nZVI alleviated oxidative stress and thereby significantly decreased EPS secretion [18]. These results indicate the different role of iron in the production of EPS by microorganisms. A previous study reported that biochar in combination with iron-containing substances (e.g., ferrihydrite) stimulated the production of EPS by *Shewanella oneidensis* MR-1 [33]. They attributed this effect to the fact that biochar acted as an electron shuttle to facilitate electron transfer, thereby inducing the production of EPS by *Shewanella oneidensis* MR-1. The different observations between the two studies might be due to different properties of bacteria strains used. In the treatment containing PBAT MPs alone, the contents of both protein and polysaccharide in EPS remained unchanged. However, BCP and MBC31P treatments significantly stimulated the protein secretion by *Serratia marcescens* ZY01. Furthermore, a clear decreasing trend in protein content was observed with the decrease in the iron-to-biochar ratios of MBCs. This phenomenon indicates that iron content played a critical role in modulating the protein secretion by *Serratia marcescens* ZY01 under the condition of coexposure to PBAT MPs. To date, little information is available on how the coexistence of MBCs and MPs affects the biosynthesis and secretion of EPS by bacteria. Therefore, the evidence presented here is not very sufficient to elucidate the underlying mechanisms, and further investigations are necessitated.

Environmental stress can trigger intracellular oxidative stress in microorganisms, leading to the enhanced generation of ROS [22]. Although a recent study reported that nanoplastics elevated the ROS level in microorganisms [34], the PBAT MPs in this study did not significantly alter the ROS level in *Serratia marcescens* ZY01. This discrepancy is most likely attributed to the differences in MP size and microbial species, which can influence the sensitivity of intracellular ROS production to environmental stress. Similarly, BC alone did not affect intracellular ROS levels in *Serratia marcescens* ZY01 compared with the CK treatment. However, the interaction between PBAT MPs and BC significantly induced intracellular ROS generation. This effect was primarily due to the changes in surface properties of PBAT MPs, which enhanced the environmental stress to *Serratia marcescens* ZY01 [34]. In the treatments with and without PBAT MPs, MBCs significantly elevated the intracellular ROS levels, indicating that MBCs induced oxidative stress and triggered pronounced oxidative stress responses in the cells of *Serratia marcescens* ZY01. Regarding the treatments without PBAT MPs, the highest intracellular ROS level in *Serratia marcescens* ZY01 was observed in the MBC15 treatment. This finding is likely attributed to the smaller size magnetic iron in MBC15 compared with the MBCs with higher iron-to-biochar ratios, coupled with a greater quantity of iron than in MBC17 [21], collectively exerting the strongest stress on this bacterial genus. In the treatments containing PBAT MPs, the MBC31P and MBC21P treatments induced the highest intracellular ROS generation. This is likely because the higher magnetic iron content in the two MBCs contributed to the more pronounced changes in the surface properties of PBAT MPs. This interpretation can be further supported by the observation that intracellular ROS levels were generally higher in the treatments with PBAT MPs than those without PBAT MPs. Active iron has been shown to promote the oxidation of MPs, which regulates the heterogeneous aggregation behavior of MPs [35].

The accumulation of excessive intracellular ROS induces damage to the cell membrane, leading to the enhanced cell membrane permeability of *Serratia marcescens* ZY01 [34]. Aligning with the observed intracellular ROS levels, the membrane permeability of *Serratia marcescens* ZY01 in the treatments with PBAT MPs was generally higher than in the treatments without PBAT MPs, which reinforces a critical involvement of intracellular ROS in affecting membrane integrity. Furthermore, it has been reported that iron-modified biochar, for instance biochar loaded with MgFe_2_O_4_, can upregulate genes related to the cell membrane permeability of bacteria [22]. The results in this study also demonstrate that the iron-to-biochar ratio plays an important role in modulating the cell membrane permeability of *Serratia marcescens* ZY01, irrespective of coexisting PBAT MPs. This effect is likely associated with the density of active iron and the surface structure of iron on the surface of MBCs [35]. Although previous studies have reported that MPs and their leachates generally enhance the cell membrane permeability of bacteria [9,10], we observed minimal change in the cell membrane permeability of *Serratia marcescens* ZY01 when it was exposed to PBAT MPs alone. This suggests that bacterial sensitivity to MPs can vary across bacterial strains. Interestingly, the cell membrane permeability was consistently higher in the treatments containing PBAT MPs than in the treatments without PBAT MPs, with a trend that paralleled the intracellular ROS results. This observation further supports the critical role of intracellular ROS levels in regulating the cell membrane permeability of *Serratia marcescens* ZY01.

A previous study has shown that MP particle size significantly affects cell viability, with smaller particles (≤1.0 μm) often exhibiting inhibitory effects, whereas larger particles (e.g., 5.0 μm) may not [36]. The PBAT MPs in this study stimulated the viability of *Serratia marcescens* ZY01, indicating that the particle size of PBAT MPs (approximate to 150 μm) provided a suitable habitat for the growth of *Serratia marcescens* ZY01 [37]. In the treatments without PBAT MPs, BC and most MBCs (except for MBC17) also provided favorable habitats to stimulate the cell viability of *Serratia marcescens* ZY01 [38]. Moreover, the extent of this stimulation depended on the iron-to-biochar ratio, which also persisted even in the presence of PBAT MPs. As noted earlier, MBCs with 1:1–1:3 of iron-to-biochar ratios exhibited a stronger electron transfer capacity than other MBCs, which enhanced their ability to degrade organic compounds compared with other MBCs. Accordingly, the higher cell viability of *Serratia marcescens* ZY01 in the MBC11P and MBC12P treatments compared with the MBC11 and MBC12 treatments can be attributed to the increased release of carbon sources from PBAT MPs in the former treatments.

The Mantel test analysis revealed a significantly positive correlation between prodigiosin synthesis and cell membrane permeability of *Serratia marcescens* ZY01 because prodigiosin synthesis occurred on its cell membrane [30]. Prodigiosin is involved in the respiratory chain function and cellular energy metabolism, and possesses bioactive properties such as antioxidant activity [26]. Therefore, the increase in cell membrane permeability (damage of cell membrane) might trigger prodigiosin synthesis. Furthermore, the cell membrane permeability was significantly correlated with intracellular ROS levels, further confirming that the elevated intracellular ROS level promoted the cell membrane damage of *Serratia marcescens* ZY01 [39,40]. Regarding EPS composition, a strongly positive correlation between protein and polysaccharide contents aligned with their similar variation pattern across different treatments. There was a significantly positive correlation between the cell viability of *Serratia marcescens* ZY01 and the relative abundance of *Serratia* in soil, indicating that the addition of MBCs into soils with and without PBAT MPs affected the growth of *Serratia* by modulating its cell viability. As aforementioned, *Serratia marcescens* ZY01 carries the *tet* gene. Therefore, a significantly positive correlation was observed between the relative abundance of *Serratia* and absolute abundance of the *tet* gene in soil. Although EPS-associated ARGs have been found to have a higher transformation capacity than cell-free ARGs in aqueous systems [41], no significant correlation was observed between the absolute abundance of the *tet* gene and the contents of protein or polysaccharide in the studied soil treatments. The horizontal transfer of ARGs can be influenced by multiple factors, such as bacterial ion binding, oxidative stress, energy metabolism, and the expression of genes related to cell membrane permeability, DNA repair, drug resistance, and quorum sensing [8,42,43]. In this study, the increase in intracellular ROS levels not only induced prodigiosin synthesis of *Serratia marcescens* ZY01 but also contributed to an increase in the absolute abundance of the *tet* gene in soil. This indicates that the horizontal transfer of the *tet* gene may still occur at the intra-genus level in soil environments. A recent study also found that the intracellular ROS not only directly drove the conjugate transfer of ARGs but also influenced it indirectly by altering the cell membrane permeability and cell adhesion [44].

## 5. Conclusions

Although *Serratia marcescens* is a known producer of prodigiosin, its multidrug-resistant nature poses considerable risks to human health and ecological safety. This study investigated the effects of MBCs and PBAT MPs on the physiological traits of *Serratia marcescens* ZY01 and further examined their relationships with the absolute abundance of the *tet* gene in soil. The results showed that MBCs stimulated prodigiosin synthesis in *Serratia marcescens* ZY01 by mediating the electron transfer process, which could be further enhanced in the presence of PBAT MPs. In the treatments without PBAT MPs, MBCs generally reduced the protein and polysaccharide contents in EPS. MBCs also increased intracellular ROS levels due to the enhanced intracellular oxidative stress, particularly in the treatments coexisting with PBAT MPs. The rise in the intracellular ROS levels primarily led to enhanced cell membrane permeability. The presence of solid materials generally provided favorable habitats for *Serratia marcescens* ZY01, thereby enhancing its cell viability. Furthermore, the intracellular ROS levels were significantly positively correlated with the absolute abundance of the *tet* gene in soil, indicating its contribution to the horizontal transfer of *tet* at the intra-genus level. The findings will be helpful for understanding how MBCs and MPs affect the physiological characteristics of antibiotic resistance microorganisms and subsequently influence the dissemination of ARGs in soil. This knowledge is useful for developing environmental remediation strategies based on biochar–iron composites. Nevertheless, the experimental treatments for assessing bacterial physiology and *tet* gene dynamics were separately conducted in different media. Further research conducted in the integrated reaction treatments is needed to elucidate the molecular mechanisms underlying the horizontal transfer of ARGs under the exposure to MBCs and MPs.

## Data Availability

The raw data supporting the conclusions of this article will be made available by the authors on request.

## Supplementary Materials

The following supporting information can be downloaded at: https://www.mdpi.com/article/10.3390/toxics14010035/s1, Figure S1. Effects of BC/MBCs and combination with PBAT MPs on the relative abundance of *Serratia* in soil (The different lowercase letters mean significant differences between different treatments without PBAT MPs, *p* < 0.05. The different uppercase letters mean significant differences between different treatments with PBAT MPs, *p* < 0.05. The asterisks “*, **, and ***” represent the significant difference at *p* < 0.05, *p* < 0.01, and *p* < 0.001, respectively). Figure S2. Effects of BC/MBCs and combination with PBAT MPs on the absolute abundance of *tet* gene in soil (The different lowercase letters mean significant differences between different treatments without PBAT MPs, *p* < 0.05. The different uppercase letters mean significant differences between different treatments with PBAT MPs, *p* < 0.05. The asterisks “*, **, and ***” represent the significant difference at *p* < 0.05, *p* < 0.01, and *p* < 0.001, respectively).
