# Supplementary material for: Physiological Responses of Serratia marcescens to Magnetic Biochars and Coexisting Microplastics and the Relationships with Antibiotic Resistance Genes
Source: Toxics. 2025 Dec 28;14(1):35. doi: 10.3390/toxics14010035 (PMC12845968; doi:10.3390/toxics14010035)
Supplement: Supplementary file 1 [file toxics-14-00035-s001.zip › toxics-3994756-supplementary.pdf]

Physiological responses of *Serratia marcescens* to magnetic biochars and coexisting microplastics and the relationships with antibiotics resistance genes

Guixiang Zhang [1], Rui Ren [1], Xiaohui Zhang [2], Yuen Zhu [3,*], Yanxia Li [4], Long Ping [1]

[1] School of Environment and Resources, Taiyuan University of Science and Technology, Taiyuan 030024, Shanxi Province, China

[2] Engineering Research Center of Coal-Based Ecological Carbon Sequestration Technology of the Ministry of Education, Key Laboratory of Graphene Forestry Application of National Forest and Grass Administration, Shanxi Datong University, Datong 037009, China

[3] Shanxi Laboratory for Yellow River, College of Environmental & Resource Sciences, Shanxi University, Taiyuan, 030006, Shanxi Province, China

[4] State Key Laboratory of Water Environment Simulation, School of Environment, Beijing Normal University, 100875 Beijing, China

* Correspondence: zhuyuen@sxu.edu.cn (Y. Zhu)

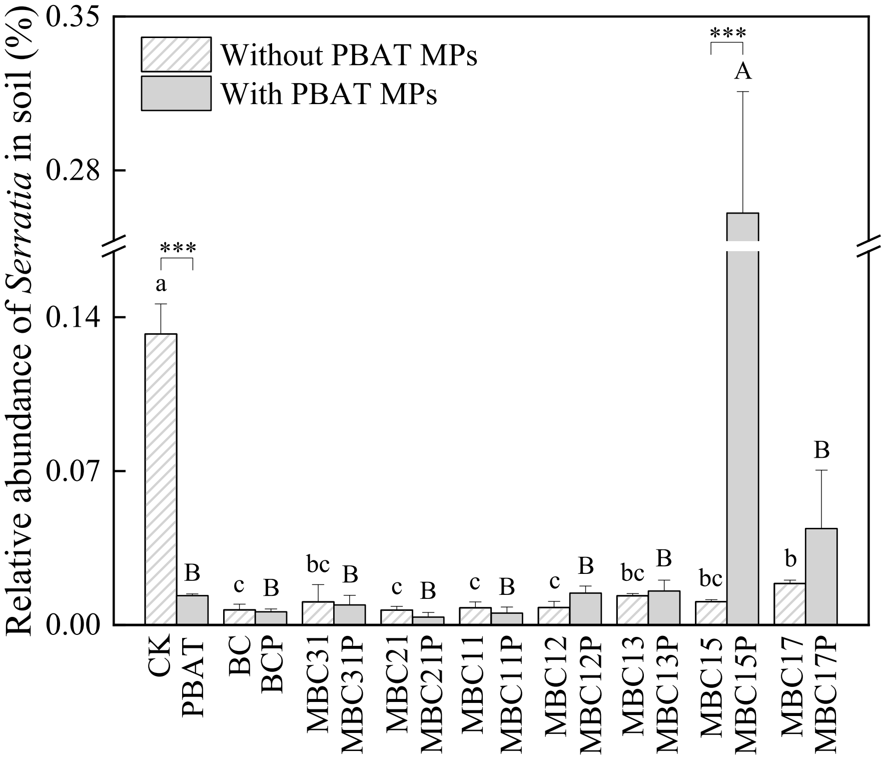

Figure S1 Effects of BC/MBCs and combination with PBAT MPs on the relative abundance of *Serratia* in soil (The different lowercase letters mean significant differences between different treatments without PBAT MPs, $p < 0.05$. The different uppercase letters mean significant differences between different treatments with PBAT MPs, $p < 0.05$. The asterisks "$*$, $**$, and $***$" represent the significant difference at $p < 0.05$, $p < 0.01$, and $p < 0.001$, respectively)

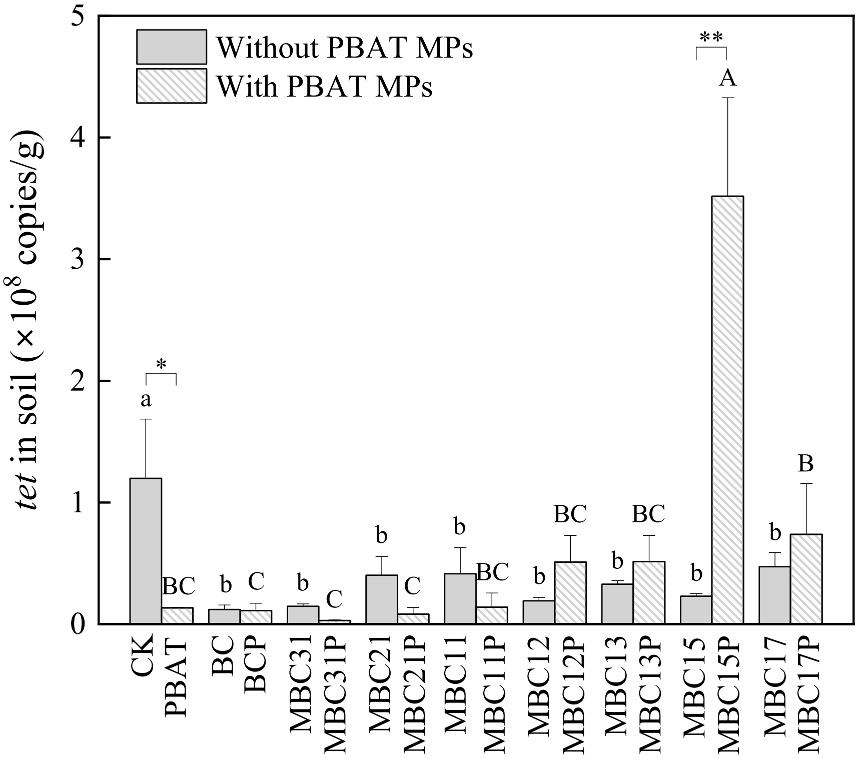

Figure S2 Effects of BC/MBCs and combination with PBAT MPs on the absolute abundance of *tet* gene in soil (The different lowercase letters mean significant differences between different treatments without PBAT MPs, $p < 0.05$. The different uppercase letters mean significant differences between different treatments with PBAT MPs, $p < 0.05$. The asterisks "*, **, and ***" represent the significant difference at $p < 0.05$, $p < 0.01$, and $p < 0.001$, respectively)
